# Soxhlet Extraction versus Hydrodistillation Using the Clevenger Apparatus: A Comparative Study on the Extraction of a Volatile Compound from *Tamarindus indica* Seeds

**DOI:** 10.1155/2021/5961586

**Published:** 2021-12-02

**Authors:** Kehinde Oluwakemi Fagbemi, Daniel Ayandiran Aina, Olufunmiso Olusola Olajuyigbe

**Affiliations:** Department of Microbiology, School of Science and Technology, PMB 4005, Babcock University, Ilisan-Remo, Ogun State, Nigeria

## Abstract

The present study aims to compare two traditional extraction techniques. A volatile compound from *Tamarindus indica* seed was obtained with Soxhlet extraction (SE) and hydrodistillation using the Clevenger apparatus (HDC). The extraction yield and chemical composition of the essential oil samples were compared. Both oils extracted were analyzed with GC-MS, and forty-one chemical compounds were identified in essential oil components from SE while forty chemical compounds were found in the HDC-extracted oil sample. The major essential oil components present in both the SE and HDC method are cis-vaccenic acid, 2-methyltetracosane, beta-sitosterol, 9,12-octadecadienoic acid (Z, Z)-, and *n*-hexadecanoic acid in varying concentrations. Moreover, the essential oils obtained by both methods look similar quantitatively but differ qualitatively. The HDC method produced more oxygenated compounds that contribute to the fragrance of the oil. The major constituents observed in the essential oil extracted by SE methods include cis-vaccenic acid (17.6%), beta-sitosterol (12.71%), 9,12-octadecadienoic acid (Z, Z)- (11.82%), n-hexadecanoic acid (8.16%), 9,12-octadecadienoic acid, methyl ester (5.84%), oleic acid (4.54%), and 11-octadecenoic acid and methyl ester (3.94%). However, in the hydrodistillation technique, the oil was mostly composed of 9,12-octadecadienoic acid (Z, Z)- (23.72%), cis-vaccenic acid (17.16%), n-hexadecanoic acid (11.53%), beta-sitosterol (4.53%), and octadecanoic acid (3.8%). From the data obtained, HDC seems to be a better method for extraction of *Tamarindus indica* essential oil compared to the Soxhlet extraction apparatus.

## 1. Introduction

The earth is endowed with varieties of medicinal plants which have been a great source of many active compounds used in pharmaceutical and food industries [1]. Medicinal plants' usage is attributed to their bioactive ingredients. Plants such as *Tamarindus indica* have been a major source of many novel compounds used for drug development [2]. Other plants with such desirable bioactive compounds for medicinal purposes include *Erythrina abyssinica*, *Aloe vera*, *Albizia coriaria*, *Azadirachta indica* [3]; *Boswellia corteri* [4], and *Oroxylum indicum* (L.) [5].

*Tamarindus indica* L. generally known as tamarind is the third largest family of flowering plants with 727 genera identified. It belongs to the subfamily Caesalpinioideae of the family Leguminosae (Fabaceae) [6, 7]. Tamarind has been extensively used for numerous indigenous medicinal purposes and contributes to food security in sub-Saharan Africa. It possesses several properties which include hepatoprotective activity, antiasthmatic activity, and laxative such as anti-inflammatory activity and antibacterial activity among others [8]. Moreover, other valuable bioproducts such as essential oil can be obtained from tamarind plant. Hence, it has been reported to be one of the most significant multipurpose trees from the tropics [9].

Essential Oils (EOs) are composite combinations of low-molecular-weight volatile compounds extracted from plants parts such as flowers, leaves, seeds, fruits, and stems of aromatics plants [10]. Essential oils, among other usage, can be used to prolong fruit shelf life, thus maintaining their postharvest quality. The major ingredients which are responsible for the distinctive aroma and biological activities of EOs are the terpenoids and phenylpropanoids [10]. Furthermore, EOs are characterized by their hydrophobicity nature. Several techniques are used for the extraction of Eos. These include supercritical fluid extraction, subcritical extraction liquid, solvent-free microwave extraction, hydrodistillation, steam distillation, hydrodiffusion, Soxhlet extraction, and solvent extraction [11]. The composition of the EOs may differ from one another depending on the extraction techniques applied. The extraction technique and the operating conditions could significantly impact on process performance and ultimately the extraction efficiency [12]. Hence, a suitable extraction technique is imperative to obtain a value-added product like essential oil. Currently, there is a scarcity of literature on the choice of extraction approach for the extraction of EOs from *Tamarindus indica* plant. This study highlights a suitable extraction method to obtain EOs from Tamarind seeds. Evidently, the *Tamarindus indica* structure becomes more accessible and solubilizes to obtain essential oil units [13]. Extraction methods that will be used in this study include Soxhlet extraction and hydrodistillation using the Clevenger apparatus.

Soxhlet extraction is one of the traditional techniques that have been extensively used for the extraction of bioactive compounds from numerous natural sources. For several decades, this technique has been consistently functional in various analytical processes in relation to the extraction of bioactive compounds [14]. One of the main advantages of the Soxhlet extraction technique is that compounds with average to low solubility can be extracted with this technique [15]. To obtain a satisfactory yield from this technique as well as to avert the loss of volatile compounds, the right choice of solvent is imperative. Also, the type of solvent used in this technique is suggestive of the polarity of the compounds pulled out. The extraction period is typically long, thereby resulting in the destruction of some thermolabile compounds [16].

Hydrodistillation is another conventional method which uses water or steam for the extraction of bioactive compounds, mostly essential oils. This technique is regularly performed via a setup recognized as the Clevenger apparatus or simple steam distillation. In the Clevenger apparatus, the hydrated sample is heated to vaporize volatile constituents, while in the steam distillation approach, steam is passed through a bed of the sample. In both methodologies, two layers (aqueous and oil-rich) are achieved and oil can be further separated via separating funnels. From economical point of view, this technique does not require the use of organic solvents [17], making it a desirable option when extraction cost is of importance. Hydrodistillation involves three main physicochemical processes: hydrodiffusion, hydrolysis, and heat decomposition [18].

In view of that mentioned above, the choice of extraction technique is critical to the viability of the volatile compound to be extracted and obtaining a desirable yield. Many extraction techniques resulted in a very low concentration of the extracted compound; therefore, it is very vital to explore the efficacy of different extraction processes to discover the most preferable technique for the extraction of essential oils from plant material such as *Tamarindus indica* [19]. There is a dearth of studies on the usage of Soxhlet and hydrodistillation conventional methods on EO extraction from tamarind seed. Therefore, the main objectives of this study are to access Soxhlet and hydrodistillation (with the Clevenger) convectional techniques for the extraction of essential oil from *Tamarindus indica* seed as well as compare the quality of the extracted essential oil using gas chromatography coupled with mass spectrometry (GC-MS) for identification of compounds present.

## 2. Materials and Methods

Tamarind fruits were purchase from a market in Michika, Yola, Adamawa state, Nigeria, in April 2019. The pulp was removed in order to get the seed. Seeds obtained were sun-dried. The dried seeds were crushed and pulverised to powder. The particle sizes range from 0.05 to 2.0 mm.

### 2.1. Preparation of Extracts

Tamarind fruits were collected from Yola, Adamawa state, Nigeria. They were botanically authenticated by a taxonomic (Dr. Nodaz George) from the University of Lagos herbarium with a voucher no. LUH: 8772 and was deposited at the herbarium. The fruits were sun-dried and peeled to collect the seeds. The moisture content of the seed was determined before pulverising. All reagents used for this study were of the highest analytical grade and were obtained from Sigma-Aldrich Chemical Company (Germany).

### 2.2. Extraction Process

#### 2.2.1. Soxhlet Extraction Method (SE)

A total of 100 g of the pulverised tamarind seeds was kept in a thimble holder before placing it into the Soxhlet apparatus. Petroleum ether (500 ml) was dispensed in a round-bottom flask to initiate the extraction process. The extraction was carried out for 6 h, at temperature between 30 and 60°C. When the solution in the thimble was clear, it signified that the oil was completely extracted from the raw seed and the apparatus was switched off. The collected solvent and the oil were placed in a water bath to remove the solvent, and the oil was golden yellowish with high viscosity [20].

#### 2.2.2. Hydrodistillation with the Clevenger (HDC)

Pulverised seeds of tamarind were subjected to hydrodistillation with the Clevenger under optimal operational conditions with a temperature of 40°C as described by Elyemni et al. [21]. One hundred grams (100 g) of pulverised tamarind seed was mixed with 800 ml of distilled water. The distillation process was performed for 3 h, and the obtained essential oil was collected and dehydrated using anhydrous sodium sulphate.

#### 2.2.3. Moisture Content

The moisture content of the seed was determined before pulverising and calculated as follows.

Dry basis moisture content (*M*_*d*_) is described by the percentage equivalent of the ratio of the weight of water (*W*_*w*_) to the weight of the dried sample (*W*_*d*_):

(1) $$\begin{array}{c} M_{d}=\left(\frac{W_{w}}{W_{d}}\right)\times 100. \end{array}$$

#### 2.2.4. Extraction Yields

For each extraction technique, the total yield is defined as gram of oil extracted per kg of the pulverised material seed into the extraction apparatus.

The extraction yields of the essential oils obtained from both methods were calculated as follows:

(2) $$\begin{array}{c} \text{Extraction yield}\left(\%\right)=\frac{\text{Mass of extracted oil}}{\text{Mass of pulverised seed}}\times 100. \end{array}$$

### 2.3. Gas Chromatography-Mass Spectrometry (GC-MS) Analysis

GC-MS analysis was carried out on GCMS-QP2010 (Shimadzu, Tokyo, Japan) mass selective detector equipped with an AOC-20i autosampler injector and fitted with a BP-20 capillary column (SGE International, Ringwood, Australia) (30 m length × 0.25 mm internal diameter; 0.25 *μ*m film thickness, polyethylene glycol, TPA treated). The samples were diluted as 10 *μ*L in 2 mL DCM (Merck KGaA, Darmstadt, Germany, HPLC grade); sample injection volume was 1 *μ*L. Helium (99.99% pure, M/s. J.K. Enterprise, Nasarala, Hoshiarpur, Punjab, India) was used as carrier gas with 1.28 mL/min flow rate, linear velocity 46.3 cm/second, column pressure: 144.4 kPa, and a split mode ratio of 15 : 1. The oven temperature was programmed as mentioned in the GC. The injector port temperature was maintained at 250°C. Significant quadrupole MS operating parameters: interface temperature, 250°C; ion source temperature, 200°C; electron impact ionization at 70 eV with 0.9 kV detector voltage, a scan mass range of 50–800 at a sampling rate of 1.0 scan/second, scan speed: 1428 u/second, and interval: 0.3 seconds.

## 3. Result and Discussion

In order to evaluate the impact of different extraction techniques on the chemical composition of EOS from tamarind seed, Soxhlet extraction and hydrodistillation with the Clevenger were used. For each extraction technique, the total yield was defined as gram of oil extracted per kg of dried pulverised seed filled into the extraction apparatus. The moisture content of the seed was determined before pulverising, and it gives an approximate value of 8.2% d.b. The techniques used are first compared in terms of the yield. It was observed that SE yielded more oil than the HDC as shown in Table 1. This could be due to the long period of extraction of SE (6 h) compared to HDC (3 h). It is noteworthy to point out the reason for the different extraction time used. For Soxhlet extraction, there are minimum numbers of cycles that are required (16 cycles), and it takes between 6 and 8 h [22]. Both methods produced a yellowish color of EOS with unpleasant odour. Extraction time is one of the important parameters usually considered for ultimate yield. This allows for sufficient degradation of the plant tissue structure and the release of adequate volatile compounds such as essential oils. Similarly, results have been reported in the literature [23].

**Figure 1:**
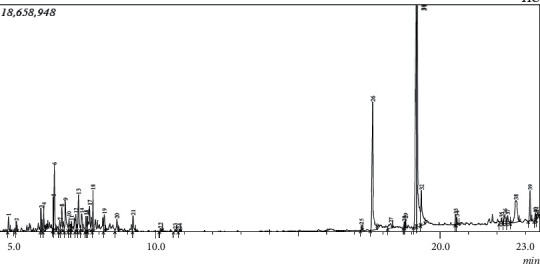
Chromatogram of essential oils of tamarind seed extracted with the Clevenger apparatus.

**Figure 2:**
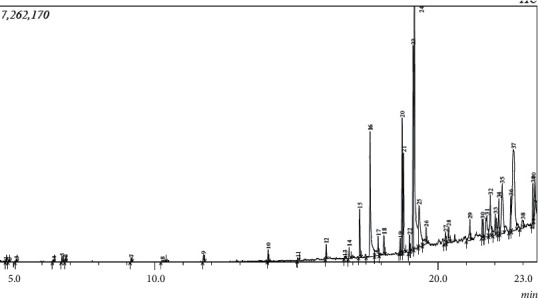
Chromatogram of essential oils of tamarind seeds extracted by Soxhlet extraction.

The compounds obtained in HDC extraction were identified early in the GC-MS quantification compared to the separation pattern obtained in the SE (Figures 1 and 2). This shows that the HDC extraction process promotes compounds with low solubility and less molecular weight while SE favours high solubility and higher-molecular-weight volatile compounds. This agrees with the report of Sermakkani and Thangapandian [24] indicating that higher-molecular-weight compounds are less volatile. Compounds with higher molecular weight require high temperature for their elution. It is interesting to know that 32 different compounds were exceptional to only the hydrodistillation method and 31 compounds were unique to the SE methods, while 6 compounds were found in both methods. Moreover, the results from Table 2 showed that the chemical constituents of the essential oils by the two methods (HDC and SE) were similar with minor quantitative variances. The predominant compounds in SE were beta-sitosterol (12.71%), oleic acid (4.54%), gamma-tocopherol (5.2%), cis-vaccenic acid (17.6%), 9,12-octadecadienoic acid (Z, Z)- (11.82%), *n*-hexadecanoic acid (8.16%), and 9,12-octadecadienoic acid and methyl ester (5.84%), while the predominant ones in HDC include beta-sitosterol (4.54%), *n*-hexadecanoic acid (11.53%), 9,12-octadecadienoic acid (Z, Z)- (23.72%), and cis-vaccenic acid (17.16). The presence of these compounds is in agreement with previous reports on *Tamarindus indica* though at different concentrations [25, 26]. Six major compounds, p-xylene, gamma-tocopherol, 2-methyltetracosane, 9,12-octadecadienoic acid (Z, Z)-, *n*-hexadecanoic acid, and beta-sitosterol, were found in both extraction processes, with beta-sitosterol having the highest proportion (12.71%) in the SE (4.53%) obtained with the HDC process. Also, the percentage of some fatty acid octadecadienoic acid (commonly known as oleic acid) quantified in SE is 4.54%, while HDC has 3.81% is noticeable variance in both methods. However, another fatty acid, cis-vaccenic acid, was present in almost the same quantity signifying it was not decomposed by heat. The presence of different proportions of beta-sitosterol and gamma-tocopherol observed in this study has been reported by other authors [27–29]. The differences in the proportion obtained could be ascribed to the duration of extraction and the extraction technique implemented.

In addition, 9,12-octadecadienoic acid (Z, Z)- was the most predominant compound in HDC with 23.72% followed by cis-vaccenic acid with 17.16% which is in agreement with the report of Mehdi et al. [30] and Abdu [26] on tamarind leaves and seed, respectively. Also, 9,12-octadecadienoic acid (Z, Z)- has been reported to possess many pharmacological potentials such as antioxidant, antimicrobial, ant-inflammatory, and hepatoprotective. Therefore, its presence in a higher quantity gives the HDC advantage over SE. Though 9,12-octadecadienoic acid (Z, Z)- was also found in SE (11.82%), it was in lower quantity.

Monoterpene hydrocarbons are not as significant as the oxygenated compounds in the aspect of good fragrance; oxygenated compounds are well known for their pleasant aroma; hence, HDC is better. However, monoterpenes are mostly used for therapeutic treatment such as antibacterial and antifungal agents. Sesquiterpenes also have the potential to be used as anti-inflammatory and anticarcinogenic agents [31].

On the other hand, different types of terpenoid at varying proportions (Table 3) were also identified. Monoterpene hydrocarbons (MHs) (0.28%), oxygenated monoterpenes (OMs) (0.14%), oxygenated sesquiterpenes (OSs) (0.145%), terpenes (Ts) (9.41%), and oxygenated diterpenes (ODs) (0.63%) were found in the SE method. Similarly, monoterpene hydrocarbons (8.2%), oxygenated monoterpenes (OMs) (2.29%), and terpenes (1.15%) were obtained in the HDC process. Notably, two terpenoids (oxygenated sesquiterpenes and oxygenated diterpenes) were observed in the SE process that were not obtained in the HDC process; however, the presence of higher-oxygenated compounds observed in the HDC method indicates the superiority of the oil obtained over SE and could be accredited to small quantity of water content in the system which would have minimized the thermal decomposition of oxygenated compounds when compared with the SE method. A major impact of heat was observed when the quantity of p-xylene was reduced from 1.75% in HDC to 0.08% in SE. This is as a result of thermal decomposition by partial dehydrogenation of the methyl groups. Petroleum ether is the best solvent used in research due to its relative cheap cost and low boiling point (30–60°C) [32], but there was no significant difference in the compounds eluted except for the terpenoids. It can be suggested that the SE techniques using petroleum ether are suitable for the extraction of terpenoid portion from tamarind plant seeds. The observed variation in the terpenoid type and proportion could be attributed to the operational temperature, solvent used, and the process duration.

Comparing the outcome of this study with some earlier report on the same plant (tamarind), there were significant differences in both qualitative and quantitative proportion obtained. These variance could be attributed to many factors including chemical reactions such as thermal degradation, allylic rearrangement, hydrolysis, and oxidation, which are instigated and heightened due to the impact of high temperatures and water content. Other factors that are likely to be responsible are the genotype of the plant, geographic location of the plant, and mode of extraction [33].

## 4. Conclusions

From the data obtained in this study, it is observed that the volatile compounds of the essential oil obtained from *Tamarindus indica* seeds depend on the method utilized for the extraction. This buttresses the fact that the chemical composition of essential oils relies on the type of extraction methods applied. The Soxhlet extraction method (SE) resulted in higher yields in terms of quantity but lower quality due to low amount of oxygenated compounds than that obtained from hydrodistillation using the Clevenger method. Some compounds identified showed no substantial differences as far as the method applied was concerned, while others produced higher contents with a particular technique. Apparently, the composition of volatiles compounds is significantly affected by extraction techniques. In conclusion, the HDC method is considered a better method for the extraction of qualitative essential oil from the seed of *Tamarindus indica*.

## Figures and Tables

**Table 1:** Percentage yield of the two methods used.

Methods used	Fresh sample (g)	Quantity yield (g)	% yield
Soxhlet	100	2.5	2.5
Hydrodistillation with the Clevenger	100	1.4	1.4

**Table 2:** Gas chromatography-mass spectrometry result of the EOS of tamarind seed obtained from the two methods.

S/N	Compounds	Formulae	MW	Sample %	
				HDC	SE
1	o-Xylene	C_8_H_10_	106	0.77	0
2	p-Xylene	C_8_H_10_	106	0.75	0.08
3	Hydrazinecarboxylic acid, phenylmethyl est	C_8_H_10_N_2_O_2_	166	-	0.06
4	3-tert-Butyl-5-chloro-2-hydroxybenzophenone	C_17_H_17_ClO_2_	288	1.29	—
5	Benzeneethanol,alpha-, beta-dimethyl-	C_10_H_14_O	150	—	0.14
6	Benzene, 1,2,4-trimethyl	C_9_H_12_	120	1.34	—
7	Benzene, 1,2,3-trimethyl	C_9_H_12_	120	—	0.16
8	Dodecane	C_12_H_26_	170	1.6	—
9	Dodecane	C_9_H_12_	120	3.08	—
10	1-Decyne	C_10_H_18_	138	—	0.2
11	1-Hexanol, 2-ethyl-	C_8_H_18_O	130	—	0.19
12	2H-pyran, tetrahydro-4-methyl-2-(2-methyl	C_10_H_18_O	154	1.04	—
13	Oxalic acid, isobutyl nonyl ester	C_15_H_28_O_4_	272	—	0.14
14	cis-3-Methylcyclohexanol	C_7_H_14_O	114	1.67	—
15	2-Oxepanone, 7-methyl-	C_7_H_12_O_2_	128	—	0.28
16	Benzene, 1,2,3-trimethyl-	C_9_H_12_	120	2.75	—
17	Tridecane	C_13_H_28_	184	—	0.3
18	Cyclohexane, (1-methylpropyl)-	C_10_H_20_	140	1.09	—
19	2-Bromo dodecane	C_12_H_25_Br	248	—	0.47
20	Benzene, 2-propenyl-	C_9_H_10_	118	0.55	—
21	Sulfurous acid, 2-propyl tridecyl ester	C_16_H_34_O_3_S	306	—	0.16
22	Benzene, 1-methyl-3-propyl	C_10_H_14_	134	1.55	-
23	Hexadecane	C_16_H_34_	226	—	0.55
24	Spiro[3.5]nona-5,7-dien-1-one, 5,9,9-trimet	C_12_H_16_O	176	2.39	—
25	Vinyl 10-undecenoate	C_13_H_22_O_2_	210	—	0.11
26	Naphthalene, decahydro-, trans	C_10_H_18_	138	1.75	—
27	1-Hexadecanol	C_16_H_34_O	242	—	0.54
28	Bicyclo[3.1.1]hept-2-en-6-ol, 2,7,7-trimeth	C_12_H_18_O_2_	194	0.93	—
29	Hexadecanoic acid, methyl ester	C_16_H_32_O_2_	256	—	1.18
30	p-Cymene	C_10_H_14_	134	0.84	—
31	*n*-Hexadecanoic acid	C_16_H_32_O_2_	256	—	8.16
32	Benzene, 1-ethyl-2,3-dimethyl	C_10_H_14_	134	2.22	—
33	Tritetracontane	C_43_H_88_	604	—	1.69
34	Undecane	C_11_H_24_	156	1.97	—
35	i-Propyl 14-methyl-pentadecanoate	C_19_H_38_O_2_	298	—	1
36	Benzene, 1,2,4,5-tetramethyl-	C_10_H_14_	134	1.11	—
37	1-Eicosanol	C_20_H_42_O	298	—	0.63
38	2,4-Dimethylstyrene	C_10_H_12_	132	0.21	—
39	9,12-Octadecadienoic acid, methyl ester	C_19_H_34_O_2_	294	—	5.84
40	2-Naphthalenol, 1,2-dihydro-, acetate	C_12_H_12_O_2_	188	1.07	—
41	11-Octadecenoic acid, methyl ester	C_19_H_36_O_2_	296	—	3.94
42	Nonanoic acid	C_9_H_18_O_2_	158	0.26	—
43	Tetradecanoic acid, 12-methyl-, methyl ester	C_16_H_32_O_2_	256	—	0.65
44	Naphthalene, 2-methyl-	C_11_H_10_	142	0.14	—
45	9,12-Octadecadienoic acid (Z, Z)-	C_18_H_32_O_2_	280	—	11.82
46	2,4-Decadienal, (E, E)-	C_10_H_16_O	152	0.16	—
47	cis-Vaccenic acid	C_18_H_34_O_2_	282	17.16	17.6
48	Hexadecanoic acid, methyl ester	C_17_H_34_O_2_	270	0.26	—
49	Oleic acid	C_18_H_34_O_2_	282	—	4.54
50	*n*-Hexadecanoic acid	C_16_H_32_O_2_	256	11.53	—
52	Phytol, acetate	C_22_H_42_O_2_	338	1.12	—
53	10-Undecen-1-al, 2-methyl	C_12_H_22_O	182	—	0.72
54	Cyclopropaneoctanoic acid, 2-[[2-[(2-ethyl	C_22_H_38_O_2_	334	0.32	—
55	7-Hexadecenal, (Z)-	C_16_H_30_O	238	—	1.02
56	9-Octadecenoic acid (Z)-, methyl ester	C_19_H_36_O_2_	296	0.42	—
57	9,12-Octadecadienoic acid (Z, Z)-	C_18_H_32_O_2_	280	23.72	
58	Hexadecanal, 2-methyl-	C_17_H_34_O	254	—	0.87
59	1,2-15,16-Diepoxyhexadecane	C_16_H_30_O_2_	254	—	2.25
60	Octadecanoic acid	C_18_H_36_O_2_	284	3.81	—
61	Undecanoyl chloride	C_11_H_21_C_lO_	204	0.9	—
62	Hexadecanoic acid, 2-hydroxy-1- (hydroxym	C_19_H_38_O_4_	330	—	1.47
63	Diisooctyl phthalate	C_24_H_38_O_4_	390	0.53	1.76
64	gamma-Tocopherol	C_28_H_48_O_2_	416	1.05	5.2
65	beta-Sitosterol	C_29_H_50_O	414	4.53	12.71
66	Cholest-22-ene-21-ol, 3,5-dehydro-6-metho	C_33_H_54_O_3_	498	—	1.02
67	cis-13,16-Docasadienoic acid	C_22_H_40_O_2_	336	1.94	—
68	Z, Z-3,13-octadecedien-1-ol	C_18_H_34_O	266	—	2.24
69	9-Octadecenoic acid (Z)-, 2-hydroxy-1-(hyd	C_21_H_40_O_4_	356	0.43	—
			Total	**98.25**	**100**

MW- molecular weight in gram, HDC- hydrodistillation with the Clevenger, SE- Soxhlet extraction.

**Table 3:** Different types of terpenoids present in each of the method used for the extraction.

Terpenoids	SE	Area %	HDC	Area %
Monoterpenes hydrocarbons (MHs)	p-Xylene			
	0.08	o-Xylene	1.15	
	1-Decyne	0.2	p-Xylene	1.75
			Cyclohexane,(1-methylpropyl)-	0.84
			Benzene, 1-methyl-3-propyl-	2.22
			Naphthalene, 2-methyl-	1.11
			Naphthalene, decahydro-, trans-	1.21

Oxygenated monoterpenes (OMs)	Benzeneethanol, alpha-, beta-dimethyl-	0.14	2,4-Decadienal, (E, E)-	
	0.16			
			Cyclohexane, (1-methylpropyl)-	1.09
			2H-pyran, tetrahydro-4-methyl-2-(2-methyl	1.04

Oxygenated sesquiterpenes (OSs)	Oxalic acid, isobutyl nonyl ester	0.14		
Terpenes	2-Methyltetracosane	9.41	2-Methyltetracosane	1.15
Oxygenated diterpenes (ODs)	1-Eicosanol	0.63		

## Data Availability

Data are available within the manuscript.
